# COVID-19 policy analysis: labour structure dictates lockdown mobility behaviour

**DOI:** 10.1098/rsif.2020.1035

**Published:** 2021-03-31

**Authors:** Samuel Heroy, Isabella Loaiza, Alex Pentland, Neave O’Clery

**Affiliations:** ^1^ The Bartlett Centre for Advanced Spatial Analysis, University College London, London W1 4TJ, UK; ^2^ Mathematical Institute, University of Oxford, Radcliffe Observatory Quarter, Woodstock Road, Oxford OX2 6GG, UK; ^3^ MIT Media Laboratory, Massachusetts Institute of Technology, E15–383, 20 Ames Street, Cambridge, MA 02139, USA; ^4^ ICTEAM Institute - Pôle en ingénierie mathématique, UCLouvain, Louvain-la-Neuve 1348, Belgium

**Keywords:** COVID-19, mobile device location data, human mobility, labour economics, inequality, policy analysis

## Abstract

Countries and cities around the world have resorted to unprecedented mobility restrictions to combat COVID-19 transmission. Here we exploit a natural experiment whereby Colombian cities implemented varied lockdown policies based on ID number and gender to analyse the impact of these policies on urban mobility. Using mobile phone data, we find that the restrictiveness of cities’ mobility quotas (the share of residents allowed out daily according to policy advice) does not correlate with mobility reduction. Instead, we find that larger, wealthier cities with more formalized and complex industrial structure experienced greater reductions in mobility. Within cities, wealthier residents are more likely to reduce mobility, and commuters are especially more likely to stay at home when their work is located in wealthy or commercially/industrially formalized neighbourhoods. Hence, our results indicate that cities’ employment characteristics and work-from-home capabilities are the primary determinants of mobility reduction. This finding underscores the need for mitigations aimed at lower income/informal workers, and sheds light on critical dependencies between socio-economic classes in Latin American cities.

## 1. Introduction

Across the world, governments and scientists alike have struggled immensely with the question of which policies will most effectively reduce the spread of coronavirus disease 2019 (COVID-19). In a cross-country analysis of case data, Hsiang *et al.* [1] find that in six countries various policies ranging from full lockdown to paid sick leave prevented or delayed an estimated 495 million cases from January to June 2020, but that the effects of specific policies differed from country to country. Evidence from China [2,3] and across Europe [4] demonstrates clearly that full and persistent lockdown is by far the most effective measure in curbing spread. However, it is not yet clear how local economic conditions affect policy success.

Epidemic mitigation policies are not implemented in a vacuum. For instance, a tech hub has many jobs that can be performed remotely with relative ease, while a manufacturing centre does not. Globally, cities with many teleworkable jobs have been better able to reduce work commutes [5,6]. More generally, wealthier cities and wealthier neighbourhoods have been more adept at reducing urban mobility [7–10], and experienced lower COVID-19 death rates at least in the UK and USA [11,12]. However, the relationship between lockdown severity, city wealth and observed mobility reduction remains not well understood. For example, are harsh mobility restrictions as effective in less wealthy cities? Or does the nature of the local economy outweigh policy severity?

**Figure 1.**
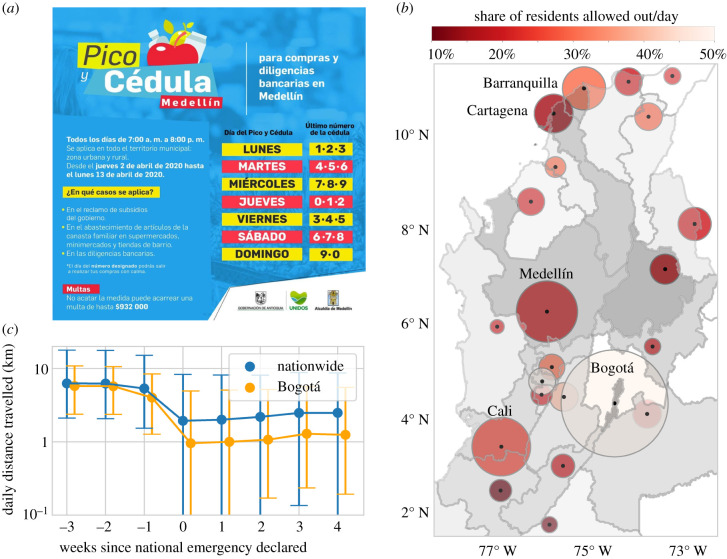
Colombian municipalities administered locally varying policies in which residents were allowed to go out for essential purposes on days corresponding to their national ID/gender. (*a*) During the first half of the study period, Medellín allowed out three ID numbers daily (30% of residents), though it later tightened the policy to only allow out two ID numbers daily (20% of residents). Image reproduced with permission from the Alacaldía de Medellín. (*b*) The capital municipalities (points, size proportional to population) of the most populous departments in Colombia, coloured by the average share of residents allowed out per weekday from 13 to 27 April. Departments are shaded by GDP *per capita*. (*c*) Users nationwide reduced their mobility greatly from 19 March (end of week −1), when the government first announced national lockdown-related policies. From 19 March to 9 April (weeks 0–2), residents nationwide were instructed to stay at home unless absolutely necessary, but localized policies took effect from 6 April (week 2) in all municipalities except Bogotá, which implemented the pico y género policy on 13 April (week 3).

Latin American countries generally had more time to prepare for the in-coming pandemic and develop appropriate policy measures [13]. In Colombia, Ecuador and Panamá, residents were allowed out for essential trips on days of the week corresponding to their national ID number and/or gender [14,15]. We can quantify the restrictiveness of this type of policy via, for example, the share of residents allowed out daily. We specifically focus on Colombian cities which implemented local variations of these policies. For example, in Bogotá residents were allowed out every other day based on their gender, whereas Florencia allowed just 10% of residents out daily (figure 1). Additionally, socio-economic conditions vary significantly between Colombian cities, with the largest cities having much higher composite wealth and industry sophistication [16,17]. These variations in policy and wealth represent a natural experiment in which we examine the relationship between local policy, city wealth and observed mobility reduction.

We use mobile phone data (call records) to characterize changes in city residents’ urban mobility in an 11 day period beginning with the introduction of local lockdown measures. Urban mobility metrics from mobile phones have frequently been used to quantify mobility reductions in the wake of COVID-19 as well as other infectious diseases, both to characterize lockdown measures [2,18] and to predict epidemic spread [19–22].

Our key finding is that more severe mobility quotas have no significant impact on local mobility reduction levels. An implication of this finding is that fine-tuned restrictions, which calibrate the share of people allowed out daily, do not lead to a proportional decrease in mobility. On the other hand, city size is strongly correlated with mobility reduction, in terms of both trip frequency and daily distance travelled. Hence, cities with higher labour formality, gross domestic product (GDP) *per capita*, and industrial complexity experience more reduced mobility—irrespective of the quota imposed. Furthermore, within cities, commuters to wealthy and commercial areas are most disrupted. Hence, both high-income and associated service workers are more likely to stay at home. Taken together, these results have important implications for the design of lockdown policies, the success of which depends critically on local economic conditions.

## 2. Methods

### 2.1. Data

#### 2.1.1. Municipal policy details

We collected (Spanish language) ‘pico y cedúla’/‘pico y género’ [14,15] policy advice from official municipal websites and social media accounts for the municipalities of interest in order to compute mobility quotas. Generally, municipal advice specified days for which local residents with ID numbers ending in certain digits were allowed out (exceptions were made for key workers). In some cases, gender (or both gender and ID) was used to differentiate residents’ allowed days out.

In order to determine the share of residents allowed out daily (mobility quota), we make simple assumptions—that each municipality is equally represented by gender (noting that gender aspects of mobility have been studied using mobile phone data in Chile [23] and in Panamá [24] during the COVID-19 pandemic) and that ID numbers are uniformly distributed (i.e. that residents in each municipality are equally likely to have an ID ending in any digit). With these assumptions, we first calculate the share of residents allowed out every day in late March/early April and then take the weekday average for the study period (13–27 April). We identify this average as the mobility quota allowed_*m*_.

In some cases, municipal advice included information regarding curfews (toqué de queda) or days segmented into times at which residents were allowed out. Also, some municipalities provided other information—including car-based restrictions (pico y placá) or information regarding where ‘pico y cedúla’ would be implemented (e.g. at grocery stores and/or public transit). Finally, municipalities differed in weekend-based restrictions—in some municipalities, the local government administered a stay-at-home order on the weekend while in others there were either special restrictions for weekends or the same as those during the week. In accord with many other studies of both general mobility patterns [25,26] and social distancing [8,9], we focus our analysis on (more regular and predictable) weekday mobility patterns and therefore weekday restrictions as well.

#### 2.1.2. Mobility data

Call detail record (CDR)-extracted mobility measures correlate well with measures from other data sources [27]. While extended detection and response (XDR)/GPS-based mobile phone data have more spatio-temporal accuracy, we use CDRs in part because of the wide penetration of our dataset throughout Colombia and in part because CDRs are reflective of users of both smartphones and ordinary cell phones. Although every mobility dataset may be subject to some bias [28], XDR/GPS-based data are likely to have more socio-economic bias (without careful control) especially in a middle-income country as such data typically come exclusively from smartphones (in 2019, smartphone penetration in Colombia was only 59% [29]).

Our analysis relies on mapping individuals to spatial units—the granularity of these units is important to the precision of our analysis. Generally, tower density varies according to municipal population, with some pairs of towers in densest cities being very close together. Pinpointing user location to very precise geographic locales (e.g. a single tower) with CDRs is generally difficult for a variety of both behavioural and technical (e.g. load-sharing) reasons, while accuracy may be enhanced by pinpointing to a more aggregate level [30,31]. We perform an agglomerative clustering algorithm [32] to join tower-associated Voronoi polygons with centroids that were within 100 m of one another. Then, we use these clusters to perform a (more aggregate) Voronoi tessellation on tower cluster centres and associate each tower with its enclosing polygon.

#### 2.1.3. Home/work detection and active user selection

Our analysis relies on having accurate estimations of home/work cell location, as well as spatio-temporal resolution of user location. Applying more stringent criteria for home/work cell detection and activity levels (activity refers to the frequency with which users make calls or receive in-network calls), however, reduces the sample size of our data and therefore the statistical power. Here, we describe the criteria that we use to transform raw CDRs to panel data. First, we expect to more accurately estimate true mobility levels for more active users—hence, we limit our subset to users who are active on a majority of weekdays during the lockdown period and at least six during a ‘basal period’ (1 January–15 March).

There is considerable work on home cell detection from CDRs [25,30,33,34]—recent work suggests that identification is most robust when regularity of night-time location is used as a criterion and tower clustering is used [30]. Here, we identify a user’s home by the cluster cell in which they are most consistently most active during night-time hours (here 0.00–7.00/22.30–24.00). Specifically, we identify a home cell if the user registers this cell as their most weeknight active night-time cell in at least three weeks during the basal period and a plurality of weeks during 16 March–30 April (inclusive of our lockdown study period). While it is commonplace to use night-time location to define home cells (e.g. [25,33]), we focus on the consistency of users' detected night-time location across weeks as our calculations depend on users keeping the same home cell during the basal/lockdown periods. This criterion is especially important here because it has been observed that many people have moved residence before/during lockdown [35]—residential movement during the study period would tend to inflate lockdown mobility.

Additionally, the problem of work cell location is quite commonplace. We identify work cells in an analogous manner to our home detection approach [25]. We identify a user with a work cell if that cell is their most common day-time (8.30–18.00) calling location on a plurality of the weeks in the basal period, and if they are active in the work cell on at least 20% of their active days in this period.

With these identifications, we transform location data from raw CDRs to panel data, in which we have estimated the home location as well as mobility levels before/during lockdown for 15 347 users across 22 municipalities. There are roughly 1 million unique users across 2020 in the raw CDR dataset, but many of these are not sufficiently active (especially during the lockdown period), fail to register a sufficiently consistent home cell and/or are not based in one of the municipalities of interest.^1^ For 9069 of the 15 347 users, we also estimate a pre-pandemic work cell that is unique from their home cell (in the other cases, either we do not detect a work cell or the work cell and home cell are the same).

#### 2.1.4. Socio-economic stratum

The national government surveys urban residential dwelling conditions to assign socio-economic strata [36]. These data are provided in the form of survey indices mapped to geographical areas. Stratum tends to be highly spatially correlated owing to socio-economic segregation. We first compute average strata for the second most granular designation—‘secciónes urbanos’ (urban sections), and then we use geographically weighted spatial averaging to assign strata to our tower cluster cells. Finally, we use *k* = 2 nearest-neighbour averaging [37] to assign stratum to clusters that do not intersect urban sections (see electronic supplementary material, figure S2). The distribution of stratum estimations across users is depicted in electronic supplementary material, figure S1B.

#### 2.1.5. Municipality-level demographic/economic variables

In addition to the cell-level socio-economic stratum, we use municipality-level official estimations of (a) population, (b) nominal GDP per capita, (c) labour formality, and (d) economic complexity. Estimates of (a) and (b) are available from DANE [36] at https://www.dane.gov.co/index.php/estadisticas-por-tema/demografia-y-poblacion and https://dane.maps.arcgis.com/apps/MapSeries/index.html?appid=71f231f4e31a40ec8796d559544e9103. For (c), we use the same very simple methodology as in [17]. That is, we simply associate labour formality with the (2017) count of formal employees in the municipality [38] divided by the working age (15–64) population [36]. This approach of course suffers from a number of limitations—(i) some of these employees will be based outside the municipality; (ii) a share of workers will be outside the working age population; and (iii) not everyone in the working population is in the workforce. Despite these limitations, it is a commonly used methodology to estimate labour formality and we expect that none of these assumptions will overwhelmingly bias our estimations for labour formality in any particular municipality. Finally, for (d) we use municipality-level industrial employment data [38], and then compute economic complexity using the method of Hidalgo *et al.* [17,39,40].

### 2.2. Mathematical modelling

In general, our mathematical modelling relies on individual-level regression analyses, though we additionally perform certain aggregate analyses as well (results are generally comparable). Where appropriate, we use a weighted approach so that all municipalities in our sample carry equal weight in our regressions. Because our response variables generally obey complex distributions, we generally use a regression bootstrapping scheme to generate confidence intervals, *p*-values and standard errors. A detailed enumeration of our methodology can be found in electronic supplementary material, section S1. Generally, we use the subscript *i* for individuals in our dataset and the subscript *m*(*i*) to denote the city to which a resident belongs (for municipality-level variables) as well as *c*(*i*) to denote the cell to which a resident belongs (for cell-level variables). We use the subscripts *m* and *c* to indicate municipality/cell-level variables (table 1).

**Table 1.** List of variable names.

trip frequency_*i*_	% of weekdays in which a user makes a trip of ≥1 km
daily dist_*i*_	weekday-average maximum distance from home cell in which user *i* is observed
disrupted_*i*_	indicator of commute disrupted for user *i* (=1 if their commute is disrupted)
allowed_*m*_	% of residents allowed out on average in a day in municipality *m*
pop_*m*_	population of municipality *m*
strat_*c*_	socio-economic stratum of cell *c*
complex_*m*_	industrial complexity of municipality *m*
formal_*m*_	formality rate of municipality *m*
GDP_*m*_	GDP *per capita* of municipality *m*
cell size_*c*_	area in square km of cell *c*
formal_*c*_	indicator of whether cell *c* ∈ Bogotá is formal
commute dist_*i*_	distance between home/work cells of commuter *i*
*N*	number of individuals in sample
*M*	number of municipalities in sample
*N*_*m*_	number of individuals in municipality *m*

## 3. Results

### 3.1. ID and gender-based mobility restrictions

During the early stages of COVID-19 exposure, the Colombian government ordered municipal governments to impose local mobility restrictions. Most municipalities (22 departmental capitals) imposed ‘pico y cédula’ restrictions in which residents were allowed out on certain days corresponding to the terminating digit of their national ID numbers. However, the ID numbers allowed out daily varied between municipalities (figure 1*a*). For example, two of the smallest municipalities only allowed residents out roughly one weekday per every two weeks (10% of residents allowed out daily). The largest city and capital, Bogotá, allowed males/females out on alternating days (50% allowed out daily).

In order to quantify the restrictiveness of municipal policies, we collected local policy advice for the principal departmental capitals from government websites and/or social media. Whereas these ID/gender-based measures were advertised widely, and enforced in certain venues (e.g. public transit, access to banks/supermarkets), the extent of policy enforcement/adherence is generally unclear across municipalities, especially in the informal labour sector.

For each departmental capital, we computed the average percentage of residents allowed out daily (or mobility quota) for 11 weekdays (13–27 April) in the pandemic’s early stages (figure 1*b*). This corresponds to an initial ’lockdown’ period in which all municipalities had recently enacted ID/gender-based restrictions. Interestingly, on average smaller municipalities permitted fewer people out daily (Pearson’s *ρ* = 0.43, *p* = 0.04). Hence, with strong adherence, we would expect that residents of smaller municipalities travelled less frequently during lockdown. In the following section, we will investigate whether this is observed in practice.

### 3.2. Effects of mobility quota and city size on lockdown mobility

In order to evaluate the effects of localized mobility restrictions, we computed mobility indicators derived from CDRs for 22 out of 23 of Colombia’s departmental capital municipalities (omitting one on account of low sample size). For each resident, we compute *trip frequency*, which is the percentage of active days (days in which the user makes or receives a call) in which they travel 1 km from their home cell, and *daily distance travelled*, which is the average distance travelled from their home cell on active days. In order to assess changes in mobility relative to pre-lockdown, we also compute these metrics for a baseline period prior to lockdown and compute change measures.

**Figure 2.**
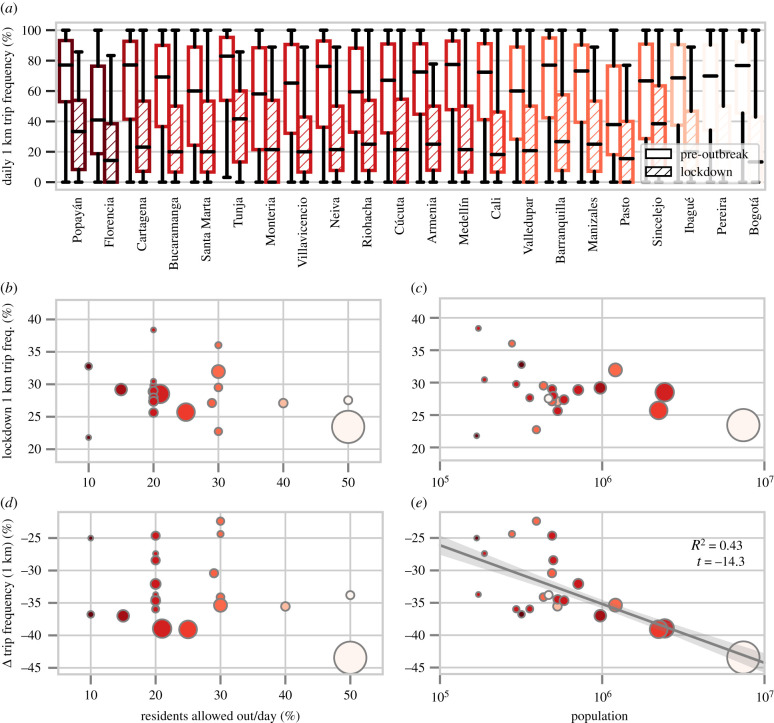
City size, rather than mobility quota severity, is associated with a more pronounced reduction in residents’ trip frequency. (*a*) Empirical distributions of trip frequencies for detected residents in municipalities of interest (ordered by quota). (*b*,*c*) Municipality-level mean trip frequency (points, size proportional to population, colour to mobility quota) during lockdown is not related significantly to quota or population. (*d*,*e*) Increasing population (but not mobility quota) has a strong reducing effect on the municipality-level median reduction in trip frequency (standard error bars and 95% confidence intervals calculated via bootstrapping).

**Figure 3.**
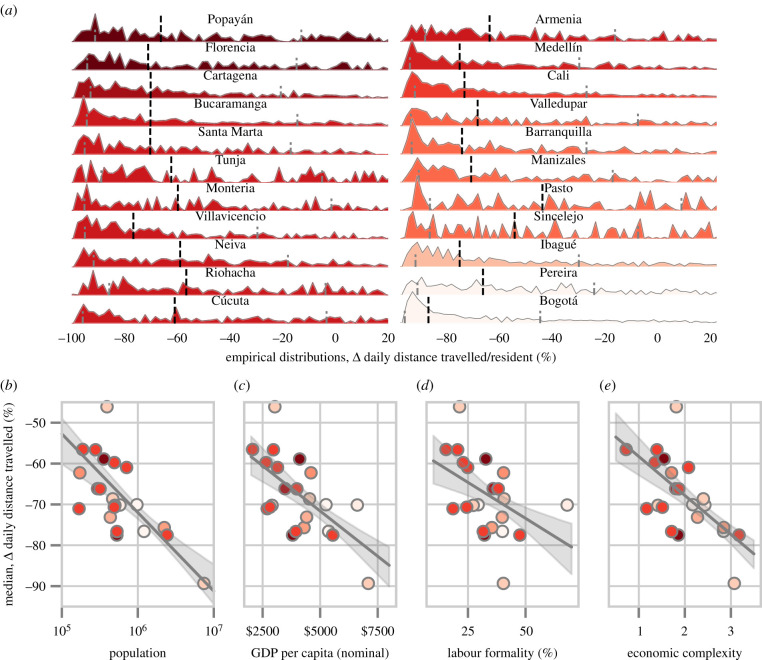
Residents of larger, wealthier municipalities with more formalized labour experienced more pronounced reductions in daily distance travelled during the lockdown period. (*a*) Empirical (kernel density) municipality-level distributions of relative change in daily distance travelled from the basal period to lockdown. Vertical dashed black (grey) lines represent municipality-level median (quartile) estimates. Municipalities are coloured/ordered by mobility quota (as in figure 2). (*b*–*e*) Municipality population, as well as economic complexity, GDP *per capita* and labour formality are associated with more pronounced relative change in daily distance travelled.

We find that most users across all municipalities reduced their daily distance travelled during lockdown (figure 1*c*). However, the extent of the reduction varied greatly across municipalities. We display municipality-level distributions of trip frequencies before and after lockdown in figure 2*a*, and relative change (from baseline) in daily distance travelled in figure 3*a*. Most municipalities had a mean lockdown trip frequency of 25–30% (figure 2*b*,*c*). This represents a reduction in mobility from baseline levels that were 20–45% higher, with the largest reduction in Bogotá (figure 2*d*,*c*).

To what extent does mobility quota restrictiveness impact *changes* in trip frequency and daily distance travelled relative to baseline levels? We find no evidence for an effect on daily distance travelled and statistically insignificant evidence (*p* > 0.1) for an effect on reduction in trip frequency. If a municipality reduced the amount of residents allowed out daily by 1%, residents on average decreased their trip frequency by only a meagre additional 0.04% (controlling for population; see table 2*a*,*b* for detailed results).

**Table 2.** Individual/municipality-level regression estimates for effects of mobility quota and city size on lockdown behaviour. Statistical significance is indicated by asterisks. Parentheses indicate standard error estimates.

dep. variable: Δ trip frequency_*i*_				
ind. variable	(i)	(ii)	(iii)	(iv)
(*a*) *individual-level effects*				
allowed_*m*(*i*)_	−0.12***		0.04	0.04
	(0.02)		(0.03)	(0.03)
log (pop_*m*(*i*)_)		−9.07***	−9.49***	−9.13***
		(0.63)	(0.77)	(0.83)
interaction_*m*(*i*)_				−0.25
				(0.16)
const	−29.97***	19.25***	20.74***	18.64***
	(0.67)	(3.79)	(4.21)	(4.65)
*N* (individuals)	15 347	15 347	15 347	15 347
*R*^2^	0.002	0.018	0.018	0.018

dep. variable: mean (Δ trip frequency_*i* ∈ *m*_)				
ind. variable	(i)	(ii)	(iii)	(iv)
(*b*) *aggregate effects*				
allowed_*m*_	−0.12***		0.04	0.04
	(0.02)		(0.03)	(0.03)
log (pop_*m*_)		−9.07***	−9.49***	−9.13***
		(0.64)	(0.79)	(0.86)
interaction_*m*_				−0.25
				(0.16)
const	−29.97***	19.25***	20.74***	18.64***
	(0.67)	(3.84)	(4.31)	(4.77)
*M* (municipalities)	22	22	22	22
*R*^2^	0.05	0.43	0.43	0.44

dep. variable: relative Δ daily dist_*i*_				
ind. variable	(i)	(ii)	(iii)	(iv)
(*c*) *individual-level effects*				
allowed_*m*(*i*)_	−0.3***		−0.1***	−0.1**
	(0.0)		(0.0)	(0.0)
log (pop_*m*(*i*)_)		−17.1***	−15.8***	−14.8***
		(0.9)	(0.9)	(1.1)
interaction_*m*(*i*)_				−0.4**
				(0.2)
const	−60.5***	29.4***	24.2***	18.4***
	(1.0)	(5.3)	(5.0)	(6.2)
*N* (individuals)	15 347	15 347	15 347	15 347
pseudo *R*^2^	0.001	0.002	0.002	0.003

dep. variable: median (relative Δ daily dist_*i* ∈ *m*_)				
ind. variable	(i)	(ii)	(iii)	(iv)
(*d*) *aggregate effects*				
allowed_*m*_	−0.3***		−0.1	−0.0
	(0.1)		(0.1)	(0.1)
log (pop_*m*_)		−17.7***	−17.2***	−15.9***
		(2.6)	(3.0)	(3.3)
interaction_*m*_				−0.9
				(0.6)
const	−59.4***	34.5**	32.6*	24.8
	(2.4)	(15.6)	(16.8)	(19.3)
*M* (municipalities)	22	22	22	22
*R*^2^	0.12	0.49	0.49	0.51

***p < 0.01, **0.01 ≤ *p* < 0.05, *0.05 ≤ *p* < 0.1.

This finding—that more severe mobility quotas do not result in proportional reductions in mobility—is unexpected and important, especially considering the breadth of findings on the role of mobility restrictions in controlling infection spread [2,4,41]. We hypothesize that urban residents are more influenced by their economic capacity to comply with rules than by the precise measures implemented locally. As a first step, we investigate the role of urbanization (city size) in mobility reduction. An abundance of previous work has shown that city size is associated with economic prosperity—larger cities boast higher wages and GDP *per capita*, and a higher skilled as well as more formalized labour force working in more sophisticated sectors [16,17,42–46]. These factors generally improve residents’ capacity to work remotely and reduce their mobility [7,9,10,21,47].

We find that city size has a strong bearing on mobility reduction. Residents of larger municipalities had more pronounced reductions in trip frequency—for every 10-fold increase in municipal population, the average resident reduced their trip frequency by an additional 9.07%. Additionally, a 10-fold increase in population was associated with a 17.1% additional reduction in daily distance travelled relative to the basal period. Moreover, when we aggregate to the municipality level, we find that city size explains nearly half of inter-municipality variance in both cases with *R*^2^ = 0.43 for change in trip frequency (figure 2*e* and table 2*b*) and *R*^2^ = 0.49 for relative change in daily distance travelled (figure 3*b* and table 2*d*).

Hence, there is something about larger cities that better enables residents to reduce mobility despite less severe mobility quotas. This result may help to explain recent findings that initial COVID-19 growth is slower on a *per capita* basis in larger cities [48]. We also find that aggregate levels of mobility reduction are significantly associated (*p* < 0.01) with municipality-level economic variables including GDP *per capita* and labour formality rate (figure 3*d*,*e* and table 3), defined as the ratio of formal workers to the working age population [17]. We construct a metric of industrial complexity [17,39,40], which is also, as expected, associated with reduction in daily distance travelled. We standardize these variables in order to facilitate comparison, and display coefficient estimates ordered from left to right in table 3 (we also display results for unstandardized versions of these regressions as well as Spearman correlations in electronic supplementary material, section S3.1). We find generally mixed results as to which of these (highly cross-correlated) variables best predict mobility reduction. While population is associated with the highest magnitude standardized coefficient estimate among all predictors for *relative* Δ *daily dist. travelled*, GDP *per capita* has the highest in the case of Δ *trip frequency*. However, for both mobility metrics, the confidence intervals for the predictor coefficients are mutually overlapping. More generally, it seems that the economic advantages of larger cities favour mobility reduction and so we next investigate the effects of more granular (neighbourhood-level) socio-economic variables on mobility reduction.

**Table 3.** Effects of standardized economic variables on lockdown behaviour.

dep. variable: mean (Δ trip frequency_*i* ∈ *m*_)				
ind. variable	(i)	(ii)	(iii)	(iv)
(*a*)				
$\tilde{{\mathrm{GDPpc}}_{m}}$	−4.13***			
	(0.26)			
$\tilde{{\mathrm{complex}}_{m}}$		−3.94***		
		(0.26)		
$\tilde{\log({\mathrm{pop}}_{m})}$			−3.50***	
			(0.24)	
$\tilde{{\mathrm{formality}}_{m}}$				−3.46***
				(0.29)
const	−32.89***	−32.89***	−32.89***	−32.89***
	(0.31)	(0.31)	(0.31)	(0.31)
*M* (municipalities)	22	22	22	22
*R*^2^	0.60	0.54	0.43	0.42

dep. variable: median (relative Δ daily dist_*i* ∈ *m*_)				
ind. variable	(i)	(ii)	(iii)	(iv)
(*b*)				
$\tilde{\log({\mathrm{pop}}_{m})}$	−6.9***			
	(1.0)			
$\tilde{{\mathrm{complex}}_{m}}$		−5.9***		
		(0.9)		
$\tilde{{\mathrm{GDPpc}}_{m}}$			−5.7	
			(0.9)	
$\tilde{{\mathrm{formality}}_{m}}$				−4.0***
				(1.1)
const	−67.5***	−67.5***	−67.5***	−67.5***
	(1.2)	(1.2)	(1.2)	(1.2)
*M* (municipalities)	22	22	22	22
*R*^2^	0.49	0.37	0.33	0.17

$\tilde{\mathrm{Variable}}$ is used to indicate that the enclosed variable is standardized across its distribution.

### 3.3. Intra-city variation by socio-economic status

City size has a clear effect on mobility reduction, but—especially in Latin America—cities themselves are characterized by high internal income inequality [49]. Here we investigate how this drives mobility reduction at an intra-city level. Specifically, we ask if wealthier residents experienced higher levels of mobility reduction.

We use the *stratum* number assigned to each residential block as a proxy for residents’ socio-economic status [36]. Stratum is a government-assigned designation corresponding to local housing conditions and quality, and has been frequently used to characterize socio-economic status [26,50]. The stratum system encompasses six strata of progressively increasing socio-economic status, with one signifying poor quality, often informal housing, and six signifying the richest neighbourhoods (stratum 6 is only present in select cities). Most residents countrywide live in medium stratum (2/3) housing.

**Figure 4.**
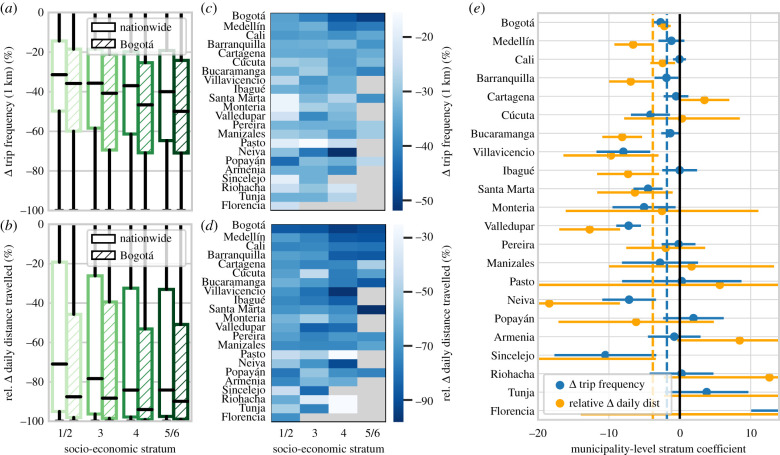
Within municipalities, higher socio-economic status was generally associated with more pronounced mobility reduction. (*a*,*b*) Boxplot distributions of residential change in trip frequency and daily distance travelled for varying stratum. (*c*,*d*) Municipality-level median estimates of change in trip frequency/daily distance travelled for varying stratum (municipalities ordered by population). (*e*) Municipality-level coefficient estimates for the effect of stratum on changes in trip frequency and daily distance travelled (error bars represent 95% confidence intervals). Dashed lines represent average effects (under the fixed-effects model) over all municipalities.

In order to examine the effects of stratum on mobility reduction, we calculate changes in trip frequency and relative change in daily distance travelled as a function of stratum for each municipality (figure 4*a*–*d*). In general, wealthier residents experienced more pronounced reductions in both daily distance travelled and trip frequency—in line with similar trends observed in cities across Latin America, Europe, the USA and Asia [7–10,21,47]. Pooling across municipalities (and taking into account fixed effects), we find that a 1 s.d. increase in stratum was associated with a 1.82% additional decrease in Δ *trip frequency (1 km)* and a 3.8% additional decrease in *rel.* Δ *daily distance travelled* (*p* < 0.01 in both cases; estimates are reported in table 4*a*,*b* and represented by dashed lines in figure 4*e*). The importance of stratum varies across municipalities—the effect of *stratum* on Δ *trip frequency* is significant (*p* < 0.05) in 10 out of 22 municipalities, and on *rel.* Δ *daily distance travelled* is significant in 11 out of 22 municipalities (coefficient estimates displayed in figure 4*e*, tabulated in electronic supplementary material, §S3.3).

**Table 4.** Pooled/fixed effects of stratum on lockdown behaviour.

dep. variable: mean (Δ trip frequency_*i* ∈ *m*_)				
ind. variable	(i)	(ii)	(iii)	(iv)
(*a*)				
strat_*c*(*i*)_	−4.00***		−2.38***	
	(0.29)		(0.31)	
${\tilde{\mathrm{strat}}}_{c(i)}$		−1.82***		−1.82***
		(0.31)		(0.31)
FE	none	none	muni.	muni.
const	−22.92***	−32.89***		
	(0.84)	(0.31)		
*N* (individuals)	15 347	15 347	15 347	15 347
*R*^2^	0.023	0.005	0.047	0.046

dep. variable: relative Δ daily dist_*i*_				
ind. variable	(i)	(ii)	(iii)	(iv)
(*b*)				
strat_*c*(*i*)_	−6.1***		−4.5***	
	(0.3)		(0.4)	
${\tilde{\mathrm{strat}}}_{c(i)}$		−4.2**		−3.8***
		(0.4)		(0.3)
FE	none	none	muni.	muni.
const	−53.7***	−69.4***		
	(1.0)	(0.4)		
*N* (individuals)	15 347	15 347	15 347	15 347
pseudo *R*^2^	0.002	0.001	0.005	0.005

We have observed the effects of socio-economic inequality on mobility reduction at both an inter-city (previous section) and intra-city level. However, we now ask—are inter-city effects explained by differences in socio-economic composition across cities? In other words, is there some effect of city size that is not simply captured by stratum variation? In order to disentangle these effects, we split each municipality into low (1/2) and high (4/6) stratum subsets and examine the effect of city size on mobility reduction across these subsets. For both groups, we find that increasing city size is associated with a greater reduction in trip frequency and daily distance travelled. The effect is comparably pronounced across low/high stratum groups—a 10-fold increase in population is associated (*p* < 0.01) with an additional 7.14% (low)/10.61% (high) reduction in Δ *trip frequency* (table 5*a*,*b*) and an additional 14.3/23.5% reduction in *rel.* Δ *daily distance travelled* (table 5*c*,*d*).

**Table 5.** Individual-level regression estimates for effects of mobility quota and city size on stratum-subset lockdown behaviour.

dep. variable: Δ trip frequency_*i*_				
ind. variable	(i)	(ii)	(iii)	(iv)
(*a*) *low-stratum subset*				
allowed_*m*(*i*)_	−0.04		0.09**	0.10**
	(0.03)		(0.04)	(0.04)
log(pop_*m*(*i*)_)		−7.14***	−8.20***	−7.70***
		(0.93)	(1.11)	(1.22)
interaction_*m*(*i*)_				−0.36
				(0.24)
const	−30.85***	9.17	12.99**	10.01
	(1.01)	(5.53)	(6.02)	(6.78)
*N* (individuals)	6593	6593	6593	6593
*R*^2^	0.000	0.011	0.012	0.013

dep. variable: Δ trip frequency_*i*_				
ind. variable	(i)	(ii)	(iii)	(iv)
(*b*) *high-stratum subset*				
allowed_*m*(*i*)_	−0.05		0.12**	0.13*
	(0.06)		(0.06)	(0.07)
log (pop_*m*(*i*)_)		−10.61***	−11.96***	−11.70***
		(2.15)	(2.24)	(2.90)
interaction_*m*(*i*)_				−0.16
				(0.52)
const	−34.86***	25.20**	29.86**	28.28*
	(2.11)	(13.05)	(13.20)	(17.38)
*N* (individuals)	3551	3551	3551	3551
*R*^2^	0.000	0.022	0.024	0.024

dep. variable: relative Δ daily dist_*i*_				
ind. variable	(i)	(ii)	(iii)	(iv)
(*c*) *low-stratum subset*				
allowed_*m*(*i*)_	−0.2***		0.0	0.1**
	(0.0)		(0.0)	(0.0)
log (pop_*m*(*i*)_)		−14.3***	−14.4***	−10.0***
		(1.1)	(1.2)	(1.4)
interaction_*m*(*i*)_				−1.8***
				(0.3)
const	−63.1***	16.1**	16.2**	−10.4
	(1.1)	(6.3)	(6.7)	(8.3)
*N* (individuals)	6593	6593	6593	6593
pseudo *R*^2^	0.000	0.002	0.002	0.002

dep. variable: relative Δ daily dist_*i*_				
ind. variable	(i)	(ii)	(iii)	(iv)
(*d*) *high-stratum subset*				
allowed_*m*(*i*)_	−0.3***		0.3***	0.2***
	(0.0)		(0.0)	(0.0)
log (pop_*m*(*i*)_)		−23.5***	−27.8***	−31.2***
		(0.5)	(0.9)	(0.8)
interaction_*m*(*i*)_				1.1***
				(0.2)
const	−65.6***	65.2***	81.7***	104.2***
	(1.0)	(3.4)	(4.6)	(4.8)
*N* (individuals)	3551	3551	3551	3551
pseudo *R*^2^	0.001	0.009	0.009	0.010

Overall, these findings underscore the role of city size in mobility reduction. We find that this effect is not limited to any socio-economic class in particular, but is instead consistent across strata. In other words, both the city in which a person resides and the socio-economic status of their own neighbourhood influence their level of mobility reduction.

### 3.4. Disruptions to work commutes

Building on previous findings that show wealthier cities have more jobs that can be performed remotely [6,51,52], we investigate how city size and socio-economic stratum are linked to work commute disruptions. We examine whether (i) city size is linked to more commute disruptions; and (ii) whether residents’ home/work stratum is linked to their tendency to forego their commutes.

**Figure 5.**
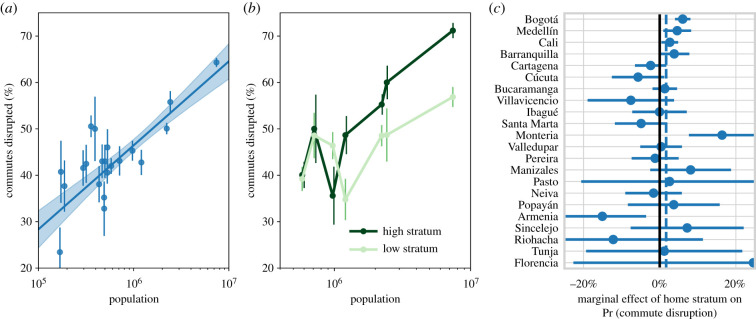
Effects of city size and socio-economic stratum on commute disruption rate. (*a*) Effect of *log(population)* on estimated *commute disruption rate* (error bars represent standard errors, 95% confidence intervals for the ordinary least squares best-fit line are calculated via bootstrapping). (*b*) Point estimates of low-/high-*stratum* subsample estimates of *commute disruption* rate for the seven largest municipalities. (*c*) Municipality-level average marginal effects of *home stratum* on the likelihood a user has their commute disrupted, with point estimates/95% confidence intervals estimated via logistic regression. Dashed vertical lines represent average effects across all municipalities, which are ordered by population.

As is standard practice, we identify home–work commutes via persistent origin–destination flows [25]. We define the commute disruption rate as the percentage of flows that cease during lockdown (see electronic supplementary material, §S2.1 for results corresponding to other definitions). As with trip frequency and distance travelled, we find, as expected, that more commute flows are disrupted in larger cities (figure 5*a* and table 6). For every 10-fold increase in population, the municipality-level commute disruption rate rises 17.8% (*p* < 0.01), while policy severity has no significant effect.

**Table 6.** Effects of mobility quota and city size on commute disruption likelihood.

dep. variable: $log\left(\frac{Pr({disrupt}_{i}=1)}{1-Pr({disrupt}_{i}=1)}\right)$				
ind. variable	(i)	(ii)	(iii)	(iv)
allowed_*m*(*i*)_	0.010***		−0.002	−0.000
	(0.002)		(0.02)	(0.003)
log (pop_*m*(*i*)_)		0.745***	0.767***	0.767***
		(0.056)	(0.062)	(0.062)
interaction_*m*(*i*)_			0.031	
				(0.027)
const	−0.586***	−4.617***	−4.694***	−4.764***
	(0.055)	(0.323)	(0.336)	(0.342)
average marginal effects				
allowed_*m*(*i*)_	0.2		−0.0	−0.0
log (pop_*m*(*i*)_)		17.8	18.3	18.3
interaction_*m*(*i*)_				0.7
*N*	9069	9069	9069	9069
LL	−6153	−6075	−6074	−6074

Using a similar approach to that of the previous section, we examine whether higher stratum residents have more disrupted commute patterns (figure 5*b*,*c* and table 7*a*). In the largest four municipalities, we find at least marginal evidence (*p* < 0.07) that increasing stratum is associated with higher commute disruption probability. However, we find significance at this level in only one other municipality, suggesting that the home stratum may play a less significant role in commute disruptions than in overall mobility reduction, particularly in smaller municipalities (the pooled average marginal effect under the fixed-effects model is 1.6%, *p* < 0.01). In these cases, other factors such as the type or location of work may also drive commute disruption.

**Table 7.** Logistic regression estimates for effect of home/work stratum on commute disruptions.

dep. variable: $log\left(\frac{Pr(disrupt_{i}=1)}{1-Pr(disrupt_{i}=1)}\right)$								
ind. variable	(i)	(ii)	(iii)	(iv)	(v)	(vi)	(vii)	(viii)
(*a*) *all commuters*								
strat_*c*(*i*,home)_	0.187***		0.079***	0.092***	0.068***		0.031	0.046*
	(0.021)		(0.023)	(0.023)	(0.025)		(0.025)	(0.026)
strat_*c*(*i*,work)_		0.311***	0.282***	0.289***		0.227***	0.222***	0.232***
		(0.021)	(0.023)	(0.023)		(0.025)	(0.025)	(0.026)
strat_*c*(*i*,home)_ × strat_*c*(*i*,work)_				−0.060***				−0.043**
				(0.018)				(0.022)
const	−0.817***	−1.209***	−1.332***	−1.365***				
	(0.059)	(0.064)	(0.074)	(.075)				
FE	none	none	none	none	muni.	muni.	muni.	muni.
average marginal effects								
strat_*c*(*i*,home)_	4.5		1.9	2.2	1.6		0.7	1.1
strat_*c*(*i*,work)_		7.4	6.7	6.8		5.2	5.1	5.3
strat_*c*(*i*,home)_ × strat_*c*(*i*,work)_				−1.4				−1.0
*N*	9069	9069	9069	9069	9069	9069	9069	9069
LL	−6126	−6054	−6048	−6042	−5953	−5915	−5914	−5912

dep. variable: $log\left(\frac{Pr({disrupt}_{i}=1)}{1-Pr({disrupt}_{i}=1)}\right)$		
ind. variable	(i)	(ii)
(*b*) *low-stratum commuters*		
strat_*c*(*i*,work)_	0.326***	0.271***
	(0.036)	(0.042)
FE	none	muni.
const	−1.255**	
	(0.106)	
average marginal effects		
strat_*c*(*i*,work)_	7.7	6.1
*N*	3360	3360
LL	−2232	−2153

While our data do not reveal the industry or occupation of commuters, we can distinguish workers according to where they work. Broadly, commuters to wealthier areas fall into two groups: they might either work at more formalized, sophisticated firms or provide services to wealthier employers or residents. In the latter case, evidence from the USA points to faster and more pronounced drops in spending among wealthier classes during the pandemic [53], corresponding to reduced demand for services in these areas. Here, we examine whether the stratum of a commuter’s destination is linked to the likelihood that they disrupt their commute. Specifically, we ask whether low-stratum commuters experienced more commute disruptions when working in high-stratum or commercial/industrial areas.

**Figure 6.**
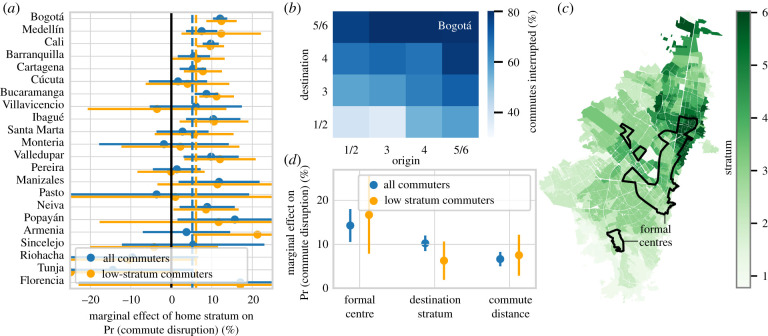
*Commutes terminating in wealthier areas and formal labour centres were more likely to be disrupted.* (*a*) Marginal effects of *destination stratum* on the likelihood a user has their commute disrupted across all detected commuters/in low-*stratum* commuter subsamples of each municipality, ordered by population (point estimates/95% confidence intervals estimated via logistic regression). (*b*) Estimates of commute disruption rate in Bogotá for varying home/work *stratum*. (*c*) Census-block characterizations of socio-economic stratum in Bogotá, with the outlined regions representing formal employment centres—zones with at least 20 000 formal employees/km^2^. (*d*) Average marginal effects of commute destination characteristics on the likelihood a commute is disrupted in Bogotá.

As expected, we find that the likelihood that a commute is disrupted depends considerably on the destination stratum (figure 6). We find a significant (*p* < 0.05) effect of destination stratum on the probability a commute is disrupted in 11 out of 22 municipalities, including six of the largest seven municipalities. These results hold across the general population as well as for the subset of low-stratum (less than or equal to 2) commuters (coefficient estimates are displayed in figure 6*a* and reported in table 7*a*,*b*)—pooled average marginal effects (under the fixed-effects model) are quite similar for both cases (5.2% for all commuters, 6.1% for low-stratum residents, *p* < 0.01 in both cases). We see this effect most clearly in Bogotá, where commutes terminating in stratum 5/6 are twice as likely to be disrupted as commutes to stratum 5/6 (figure 6*c*). These results demonstrate that both low- and high-income workers that commuted to high-income areas were much more likely to experience disrupted commutes.

Digging deeper into the characteristics of the commute destination, we use administrative formal employment data to identify commercial/industrial centres in Bogotá (figure 6*c*). These correspond to zones with at least 20 000 formal jobs/km^2^ [54]. Generally, these centres include a range of socio-economic strata, and include the central business district (Chico-Lago) and the government (La Candelaria). Controlling for commute distance, we find that commutes to these centres were on average 14.3% (*p* < 0.01) more likely to be disrupted while a one-stratum increase was associated with a 10.2% (*p* < 0.01) increase in the probability a commute was disrupted (point estimates in figure 6*d*, results are reported in table 8*a*,*b*). As above, we find comparable effects when limiting our commuter sample to low-stratum residents.

**Table 8.** Effects of work stratum along with geographical labour formalization on commute disruption likelihood in Bogotá.

dep. variable: $log\left(\frac{Pr({disrupt}_{i}=1|i\in Bogot\overset{´}{a})}{1-Pr({disrupt}_{i}=1|i\in Bogot\overset{´}{a})}\right)$				
ind. variable	(i)	(ii)	(iii)	(iv)
(*a*) *all commuters*				
strat_*c*(*i*,work)_	0.562***			0.505***
	(0.047)			(0.048)
formal_*c*(*i*,work)_		0.973***		0.705***
		(0.093)		(0.098)
log (commute distance_*i*_)			0.375***	0.327***
			(0.041)	(0.043)
const	−1.338***	0.248***	−2.526***	−4.108***
	(0.161)	(0.052)	(0.041)	(0.395)
average marginal effects				
strat_*c*(*i*,work)_	12.5			10.2
formal_*c*(*i*,work)_		21.3		14.3
log (commute distance_*i*_)			8.3	6.6
*N*	2441	2441	2441	2441
LL	−1507	−1533	−1548	−1442

dep. variable: $log\left(\frac{Pr({disrupt}_{i}=1|i\in Bogot\overset{´}{a})}{1-Pr({disrupt}_{i}=1\in Bogot\overset{´}{a})}\right)$				
ind. variable	(i)	(ii)	(iii)	(iv)
(*b*) *low-stratum commuters*				
strat_*c*(*i*,work)_	0.541***			0.293***
	(0.095)			(0.106)
formal_*c*(*i*,work)_		1.196***		0.779***
		(0.203)		(0.220)
log (commute distance_*i*_)			0.638***	0.351***
			(0.098)	(0.115)
const	−1.411***	−0.097	−5.244***	−3.914***
	(0.303)	(0.107)	(0.851)	(0.887)
average marginal effects				
strat_*c*(*i*,work)_	12.4			6.2
formal_*c*(*i*,work)_		27.3		16.7
log (commute distance_*i*_)			14.3	7.5
*N*	531	531	531	531
LL	−344	−344	−340	−328

Hence, we find that higher income workers—with more teleworkable occupations—were more likely to discontinue their commutes. However, we also observe a sharp lockdown effect for work in higher income and more commercial locations. While we would certainly expect high-income commuters to stay at home, the evidence points towards a tendency for lower income workers who work in more commercial locations (presumably in service-oriented occupations) to also stay at home. Therefore, we observe a lockdown ‘trickle-down’ effect—cities with higher income and more formalized firms have more commute disruptions not only for high-income workers, but also for low-income workers. This effect culminates in larger cities having higher levels of both commute and mobility disruption, trends that are consistent throughout our findings.

## 4. Discussion

Previous work has shown that lockdown policies are successful at reducing mobility and disease caseloads, using data from Europe [4] and China [2,3]. Here we consider whether the restrictiveness of the policy in terms of the share of people allowed out per day is correlated with the size of the reduction in mobility. We found, in accordance with the previous literature, that all cities experienced reductions in urban mobility—but there is no statistical relationship between the severity of local mobility quotas and the degree of mobility reduction. Larger, wealthier cities reduced mobility the most, even though they generally imposed less severe mobility quotas. Smaller cities, with more informal employment, did not experience a comparable reduction.

While the signal linking city size and variables capturing local economic structure to mobility reduction is strong, there are a number of other factors which are likely to have played a role. These include the degree of enforcement of the policy, the availability of economic aid to workers and firms, population density and the number of infections in the city. For example, on a national level, the government provided cash transfers to informal workers and families before and during the study period [55], in addition to a host of economic relief measures [56,57]. Future work might investigate further the role and distribution of aid in policies aimed at mobility restriction. Additionally, residents of cities with larger caseloads early in the pandemic are likely to have been more willing to lock down [58]. Although further investigation is needed, we expect to find that (as with GDP *per capita*, formality rate and industrial complexity) these factors correlate with city size and are thus consistent with our findings.

Our findings highlight the role of labour structure in cities’ abilities to reduce mobility. Less wealthy, often informal firms have less financial capacity to close operations. Workers at these firms are thus incentivized to continue working despite mobility restrictions. The example of Bogotá depicts this clearly—residents commuting to high-income/formalized work areas were as much as twice as likely to disrupt their commutes (compared with commutes to low-income areas). This result is borne out even for workers from low-income residential areas, suggesting that firm closures in these higher income/formalized areas affect both low- and high-income workers alike.

Our findings underscore the need for future policy measures to increase aid-based measures alongside or even in place of mobility restrictions. A large share of workers particularly in smaller cities—often informally employed and rarely in teleworkable occupations—cannot work from home (potentially leading to more rapid case growth in smaller cities [48]), and require substantial and sustained support, potentially leading to faster spread. While many countries encompassing middle- and high-income economies alike have developed historically unprecedented wage support schemes, the ability of low- and middle-income countries to sustain such schemes in the long term is a topic of central interest. This question’s importance is underscored by the current situation in which lower-/middle-income countries will see their vaccine stockpiles grow more slowly. As countries wrestle with how to balance reopening the economy with persistent reduction of viral spread, policymakers must consider the capacity of the non-teleworking, often informal population to adhere to lockdown measures.

## Footnotes

1 Only approximately 50 000 users register a common detected home cell for both the pre-lockdown and study period. Of/ these, approximately 17 000 have homes in the municipalities of interest—activity thresholds further limit the sample size of our panel set.

## Data accessibility

On account of privacy and ethical concerns, we do not share the individual-level data used to generate these findings. However, we include a repository of municipality-level aggregates as well as code used to generate aggregate findings at https://github.com/ils246/covid-19/.
